# Changes in the excitability of the medial parabrachial nucleus neurons during the chronic phase of pilocarpine-induced epilepsy in mice

**DOI:** 10.3389/fphar.2025.1474254

**Published:** 2025-03-18

**Authors:** Jinyu Xiao, Yinghui Gu, Chunhua Quan, Shulei Li, Jianmin Liang

**Affiliations:** ^1^ Department of Pediatric Neurology, Children's Medical Center, The First Hospital of Jilin University, Changchun, China; ^2^ Jilin Provincial Key Laboratory of Pediatric Neurology, Changchun, China; ^3^ Neuromedical Center, The First Hospital of Jilin University, Changchun, China; ^4^ Central Laboratory, The Affiliated Hospital of Yanbian University, Yanji, China; ^5^ Department of Histology and Embryology, College of Basic Medical Sciences, Jilin University, Changchun, China

**Keywords:** medial parabrachial nucleus neurons, pilocarpine, epilepsy, glial fibrillary acidic protein, neuronal activity, FosB/ΔFosB

## Abstract

**Introduction:**

Epilepsy is a common and serious brain disorder that often co-occurs with sleep disturbances. Sodium valproate, a conventional antiepileptic drug, alleviates sleep disorders in patients with epilepsy; however, the exact underlying mechanism remains unclear. The medial parabrachial nucleus is a crucial brain structure that regulates sleep-phase transitions. However, its role in pathogenesis of epilepsy remains uncertain. Therefore, we aimed to investigate whether medial parabrachial nucleus excitability is elevated during the chronic phase of temporal lobe epilepsy and whether sodium valproate could alleviate the pathological changes associated with temporal lobe epilepsy by modulating neuronal excitability in the medial parabrachial nucleus.

**Methods:**

We used the whole-cell current clamp technique to investigate the excitability of the medial parabrachial nucleus in a mouse chronic epilepsy model. To validate our findings, we utilized immunofluorescence staining and Western blotting to detect changes in the expression of FosB, a marker of neuronal activity, and glial fibrillary acidic protein (GFAP), a marker of reactive astrocyte proliferation, in the medial parabrachial nucleus during the chronic phase of epilepsy. We conducted a 28-day continuous gastric lavage of sodium valproate for antiepileptic treatment and observed changes in the excitability of neurons in the medial parabrachial nucleus neurons and the expression of FosB protein and GFAP after drug treatment.

**Results:**

We observed that medial parabrachial nucleus neurons in slices from mice that received pilocarpine stimulation fired more action potentials than those in slices from control animals that received saline. However, after treatment with sodium valproate, the number of generated action potentials decreased significantly. Immunofluorescence staining and Western blotting data on FosB and GFAP expression confirmed the increased excitability of medial parabrachial nucleus neurons and enhanced astrocyte reactivity during the chronic epilepsy phase.

**Conclusion:**

Our findings indicate an increase in the excitability of medial parabrachial nucleus neurons, along with increased reactivity of astrocytes in the chronic epilepsy model. Sodium valproate may improve the symptoms of temporal lobe epilepsy and reduce seizures by inhibiting medial parabrachial nucleus neuronal excitability. These results deepen our understanding of the pathogenesis of temporal lobe epilepsy and provide new perspectives and strategies for further research.

## 1 Introduction

Epilepsy is a chronic neurological disorder caused by abnormal and hypersynchronous neuronal activity. It affects more than 60 million people worldwide, with a prevalence of approximately 0.4%–1%, making it one of the most common neurological disorders globally (Duncan et al., 2006; Wang et al., 2016). Epileptic seizures can be primarily divided into two main categories: focal seizures and generalized seizures. Focal seizures originate from specific regions of the brain, while generalized seizures involve the entire brain and typically result in loss of consciousness. The term refractory epilepsy refers to seizures that remain uncontrolled despite the appropriate use of two or more antiepileptic drugs (Kwan and Brodie, 2010). Notably, temporal lobe epilepsy (TLE) is the most common form of refractory focal epilepsy (Allone et al., 2017; Olbrich et al., 2002). In mice with pilocarpine-induced epilepsy models, pathological changes similar to those observed in patients with TLE can occur, making this model commonly used for basic research on TLE (Cavalheiro et al., 1991; Goffin et al., 2007). Furthermore, the onset of epilepsy not only triggers abnormal neuronal activity but also leads to significant changes in glial cells. Among these changes, astrocyte proliferation is a typical and important pathological feature of epilepsy (Coulter and Steinhauser, 2015; Tian et al., 2005). Glial fibrillary acidic protein (GFAP) is regarded as a key marker of astrocyte response and proliferation following the onset of epilepsy and plays a critical role in the pathogenesis of the disorder. Numerous studies have shown that GFAP expression significantly increases after the onset of epilepsy (Chang et al., 1993). Moreover, astrocyte proliferation can lead to the elongation and thickening of cell processes, thereby promoting the formation of excitatory neural circuits, increasing neuronal excitability, and facilitating recurrent seizures (Witcher and Ellis, 2012).

Seizures may occur during sleep (Crespel et al., 2000). Patients with TLE often have abnormal sleep architecture, such as higher frequency of awakenings during the night, lower sleep efficiency, more frequent sleep-phase transitions, and reduced rapid eye movement (REM) sleep (de Almeida et al., 2003); these disturbances may lead to daytime sleepiness. The effect of sleep on epilepsy depends on the sleep phase. Specifically, seizures and abnormal electroencephalogram (EEG) discharges are more likely to occur during non-REM (NREM) sleep, especially in the N2 phase. In contrast, abnormal epileptic discharges are significantly less frequent during REM sleep (Tinuper et al., 2016). During NREM sleep, especially during the N2 stage, the EEG shows an increase in slow-wave activity, which is more likely to trigger seizures and abnormal discharges. In contrast, during REM sleep, EEG activity is faster and of lower amplitude, making it less likely to induce abnormal discharges. Moreover, sleep deprivation increases the frequency of TLE seizures (Crespel et al., 2000; Grigg-Damberger et al., 2020). Therefore, sleep is one of the most important factors that affect seizures. Notably, patients with TLE often have comorbid sleep disorders, with increased arousal after sleep and decreased REM sleep.

The parabrachial nucleus (PB) is located in the dorsolateral part of the pons and surrounds the superior cerebellar peduncle (Hashimoto et al., 2018). It mainly comprises glutamatergic, GABAergic, and enkephalinergic neurons. The PB is composed of three main subregions: the lateral parabrachial nucleus, medial parabrachial nucleus (MPB), and Kölliker–Fuse nucleus (Fulwiler and Saper, 1984). Notably, the PB is a key regulator of sleep and wakefulness. Specifically, glutamatergic MPB projections activate the cerebral cortex via basal forebrain neurons to produce wakefulness effects. In animals, damage to the MPB increases REM sleep. Therefore, the MPB is involved in the temporal regulation of sleep.

Valproic acid (VPA) is a drug commonly prescribed for these patients to ameliorate TLE-related sleep disorders (Kaur et al., 2013; Mekky et al., 2017; Nayak et al., 2015; Xu et al., 2021). VPA increases the levels of γ-aminobutyric acid (GABA) by inhibiting its metabolism. In addition, VPA can block voltage-gated sodium, potassium, and calcium channels, thereby exerting its antiepileptic effects and being used to treat focal and generalized seizures. In the present study, we hypothesized that the MPB may play an important role in the neurobiological mechanisms of TLE. To comprehensively investigate whether MPB excitability is elevated during the chronic phase of TLE, we utilized the whole-cell current clamp technique to record the electrical activity of MPB neurons and used immunofluorescence staining and Western blotting to analyze changes in the expression of FosB, a marker of neuronal activity, and glial fibrillary acidic protein (GFAP), a marker of reactive astrocyte proliferation. Additionally, we investigated, for the first time, whether VPA could alleviate TLE-related sleep disorders by modulating neuronal excitability in the MPB. This study could complement the existing understanding of sleep disturbances in TLE patients and aid in the development of new therapeutic strategies.

## 2 Materials and methods

### 2.1 Animals and surgery

We purchased 148 four-week-old male ICR mice from Liaoning Changsheng Biotechnology Co., Ltd. (license No.: SCXK (Liao) 2020-0001; Liaoning, China). The animals were housed at 22°C in separate cages with a 12-h light-dark cycle and provided unlimited access to food and water. All procedures used in this study were conducted in accordance with the international standards of animal welfare and were approved (Approval No. 20210422) by the local Committee for Animal Care Research at Jilin University. All efforts were made to use the lowest necessary number of animals and minimize their suffering.

### 2.2 Pilocarpine-induced status epilepticus model

On the first day of the experiment, the mice were injected intraperitoneally with 0.1 mg/mL scopolamine to antagonize the peripheral cholinergic response. Thirty minutes later, some mice were randomly selected for an intraperitoneal injection of 0.9% saline (saline group), whereas the remaining mice were injected intraperitoneally with pilocarpine (300 mg/kg) to induce seizures. In line with previous studies, scopolamine was administered to prevent the peripheral cholinergic effects that could influence seizure onset. Following pilocarpine administration, seizure activity was monitored using a modified Racine scale (Pitkänen et al., 2006). When seizures reached grade 4 or above and continued for 1 h, status epilepticus (SE) was confirmed. To terminate SE, all animals were then treated with an intraperitoneal injection of 1 mg/mL diazepam (4 mg/kg). Diazepam was used to stop SE and prevent the progression to potentially fatal seizures, as recommended by prior research (Mazzuferi et al., 2012; Tong et al., 2022). After the acute seizure phase, mice were observed for a “latent period” before progressing to chronic epilepsy, as is typical with pilocarpine-induced seizures. To monitor the chronic epileptic state, all animals were regularly observed for 28 days for behaviors such as convulsions, facial twitching, and other characteristic epileptic symptoms. On day 29, mice were euthanized, and seizure activity was re-evaluated using the Racine scale, ranging from stage 1 (mild facial twitching) to stage 5 (severe generalized seizures). Day 29 was selected as it represents the chronic epileptic state, allowing for the assessment of long-term changes in neuronal properties and the effects of sodium valproate treatment.

### 2.3 Experimental animal grouping and drug treatment protocol

The animals were randomly divided into three groups: (1) Saline group: Mice were intraperitoneally injected with saline and housed under standard conditions with adequate food, water, and a suitable environment. (2) Pilocarpine group: Mice were intraperitoneally injected with pilocarpine to induce epilepsy, and then housed under the same conditions as the saline group for 28 days. (3) Pilo + VPA group: Mice were intraperitoneally injected with pilocarpine to induce epilepsy and were gavaged with sodium valproate (189 mg/kg) once daily at 9 AM for 28 consecutive days. The VPA dose was based on previous studies (Binkerd et al., 1988; Watanabe et al., 2019; Wei et al., 2021).

### 2.4 Preparation of MPB slices

The mice were anesthetized via an intraperitoneal injection of 1% pentobarbital (50 mg/kg, i. p.) and rapidly euthanized. The brains from mice were immediately dissected out and submerged in an ice-cold artificial cerebrospinal fluid (ACSF), oxygenated (95% O_2_, 5% CO_2_) and containing (in mM) KCl (5), NaH_2_PO_4_·H_2_O (2.5), NaHCO_3_ (52), dextrose (20), sucrose (426), MgSO_4_ (2), and CaCl_2_ (2). Coronal brain slices (350 μm) containing MPB were prepared using a Leica microtome (VT-1200S; Leica, Wetzlar, Germany) and then transferred to a submerged oxygenated holding chamber with normal ACSF, containing (in mM) NaCl (126), KCl (2.5), NaH_2_PO_4_·H_2_O (1.25), NaHCO_3_ (26), dextrose (25), MgSO_4_ (2), and CaCl_2_ (2), at 34°C for 25 min. Slices were then maintained at room temperature (20°C–25°C) in ACSF for at least 1 h before recording. Subsequently, a single slice was transferred to a recording chamber, kept submerged at room temperature (20°C–25°C), and continuously perfused with ACSF at a rate of 2–3 mL/min.

### 2.5 Whole-cell current clamp recording

Whole-cell current clamp recordings were performed using an Axon 700 B amplifier (Molecular Devices, San Jose, CA, United States). Patch pipettes were pulled from borosilicate glass tubing using a P-97 micropipette puller (Sutter Instruments, Novato, CA, United States). The membrane and intrinsic properties of the recorded cells were measured in response to the intracellular injections of 500 m current steps at 20 pA increments up to 400 pA.

### 2.6 Immunofluorescence

Immunofluorescence staining was performed as previously described (Li et al., 2016). Mice from three groups (saline, pilocarpine, and pilocarpine + VPA) were deeply anesthetized using an overdose of carbon dioxide, and then their brains were quickly dissected out. Coronal slices containing MPB were cut at 20 μm using a freezing microtome (CM 1950; Leica) and stored at −80°C until stained. The slices were blocked in phosphate-buffered saline (PBS) containing 10% normal goat serum and 2% Triton X-100 for 1 h on a shaker (80 rpm) at room temperature (22°C). The slices were then incubated overnight at 4°C with the primary rabbit monoclonal antibody against FosB (1:200; #2251; Cell Signaling Technology) or the primary rabbit monoclonal antibody against GFAP (1:200; #80788; Cell Signaling Technology, Danvers, MA, United States of America). After washing with PBS three times for 5 min each, the slices were incubated again for 1 h at room temperature with a goat polyclonal secondary antibody against rabbit IgG [H + L] (1:200, #A10520; Life Technologies Carlsbad, CA, United States of America). The slices were then rinsed three times in PBS for 5 min each time and stained for 8 min with 4′,6-diamidino-2-phenylindole (C1005; Beyotime, Shanghai, China) to visualize the nuclei. The slices were then rinsed in PBS three times for 5 min each, mounted on glass slides, and covered with a coverslip with 75% (v/v) glycerol in 0.1 M PBS. The cells were photographed using an epifluorescence microscope equipped with a digital camera (Olympus BX51; Olympus U-RFL-T, Tokyo, Japan).

### 2.7 Western blotting

The brains were removed from the skull. Two 350 μm coronal slices of the MPB were collected using a freezing microtome (CM 1950; Leica) from Bregma −4.96 to −5.68 mm. The PBN is located lateral to the locus coeruleus and is divided into lateral and medial PBN by the superior cerebellar peduncle (SCP). We dissected the MPB tissue using a needle from the inferior and medial sides of the SCP, respectively, to avoid contamination from the lateral PBN and SCP tissues. The MPB tissue was immediately frozen in liquid nitrogen and stored at −80°C until further analysis. The MPB tissue was weighed, and 10 µL of lysis solution was added for every 1 mg of brain tissue. After the addition of the lysate, the brain tissue was mashed with a grinding rod, sonicated twice, and left to stand for 30 min. After sufficient lysis, the supernatant was removed by high-speed centrifugation at 12,000 rpm for 20 min at 4°C. The final protein concentrations were determined using a bicinchoninic acid protein assay kit according to the manufacturer’s instructions. The polyvinylidene fluoride membranes) were incubated with the primary antibody against GFAP (1:300; #80788; Cell Signaling Technology) dissolved in tris-buffered saline with Tween 20. The membranes were placed on a shaker (80 rpm) and incubated at 4°C overnight. After the incubation, the membranes were washed three times with tris-buffered saline with Tween 20 for 5 min each time. A mouse anti-GAPDH polyclonal antibody was used as a control. A horseradish peroxidase-labeled goat anti-rabbit or anti-mouse secondary antibody (1:1,000) was selected according to the corresponding host species, and then the membranes were incubated with this antibody for 2 h at room temperature, washed three times with tris-buffered saline with Tween 20 for 5 min each time, and then developed with an enhanced chemiluminescence kit. Protein bands were analyzed digitally using ImageJ analysis software.

### 2.8 Experimental data analysis

#### 2.8.1 Current clamp data analysis

The recordings were acquired using Clampfit 10.3 and Spike2 6.11. Mini-analysis was used for statistical analysis. GraphPad Prism 9 was used for data analysis and graphing. Experimental data are expressed as the mean ± standard error of the mean (mean ± SEM). Prior to selecting the appropriate statistical test, a homogeneity of variance test (e.g., Levene’s test) was performed to assess whether the data met the assumption of equal variances. If the variances were homogeneous, the neuronal firing frequency between groups was compared using one-way analysis of variance (ANOVA), followed by *post hoc* comparisons using the LSD test for pairwise comparisons between groups. If the assumption of equal variances was violated, non-parametric tests (Mann-Whitney U test) were used for group comparisons. Statistical significance was defined as P < 0.05. The effect of epilepsy on measured parameters was considered statistically significant if P values were <0.05.

#### 2.8.2 Immunofluorescence staining data analysis

The GFAP-positive area and number of FosB-positive cells were analyzed using Image-Pro Plus software. By setting a uniform threshold, we eliminated non-specific staining and performed automatic counting of positive cells. Data are expressed as mean ± SEM. A homogeneity of variance test (e.g., Levene’s test) was conducted to assess the assumption of equal variances. If the variances were homogeneous, group comparisons were made using one-way analysis of variance (ANOVA), followed by *post hoc* comparisons using the LSD test. If the assumption of equal variances was violated, non-parametric tests (Mann-Whitney U test) were used for group comparisons. Statistical significance was defined as P < 0.05.

#### 2.8.3 Western blotting data analysis

After color development using enhanced chemiluminescence, gel images were acquired, and protein bands were analyzed using ImageJ software. Data from each group were obtained in triplicate. Protein strip grayscale values were analyzed using GraphPad Prism 9. Data are expressed as mean ± SEM. A homogeneity of variance test (e.g., Levene’s test) was performed, and if variances were homogeneous, group comparisons were made using one-way ANOVA, followed by *post hoc* comparisons using the LSD test. If the variances were not homogeneous, non-parametric tests (Mann-Whitney U test) were applied. Statistical significance was defined as P < 0.05.

## 3 Results

### 3.1 Pilocarpine increases MPB neuronal activity during the chronic phase of epilepsy in mice

The pilocarpine mouse model is well-established as a reliable model of TLE. Mice exhibiting grade IV seizures or higher, up to status epilepticus (SE), were selected for the study. The chronic phase of epilepsy was initiated upon the first occurrence of spontaneous seizures. Following pilocarpine injection, the animals displayed behavioral changes that were consistent with previous reports (Yang et al., 2019), and their behavior was closely monitored for 4 weeks after SE. Spontaneous recurrent seizures (SRS) were first observed in the pilocarpine-treated mice starting 3 days after SE. All animals that survived SE developed SRS of stage IV or V during the subsequent 24 days.

**FIGURE 1:**
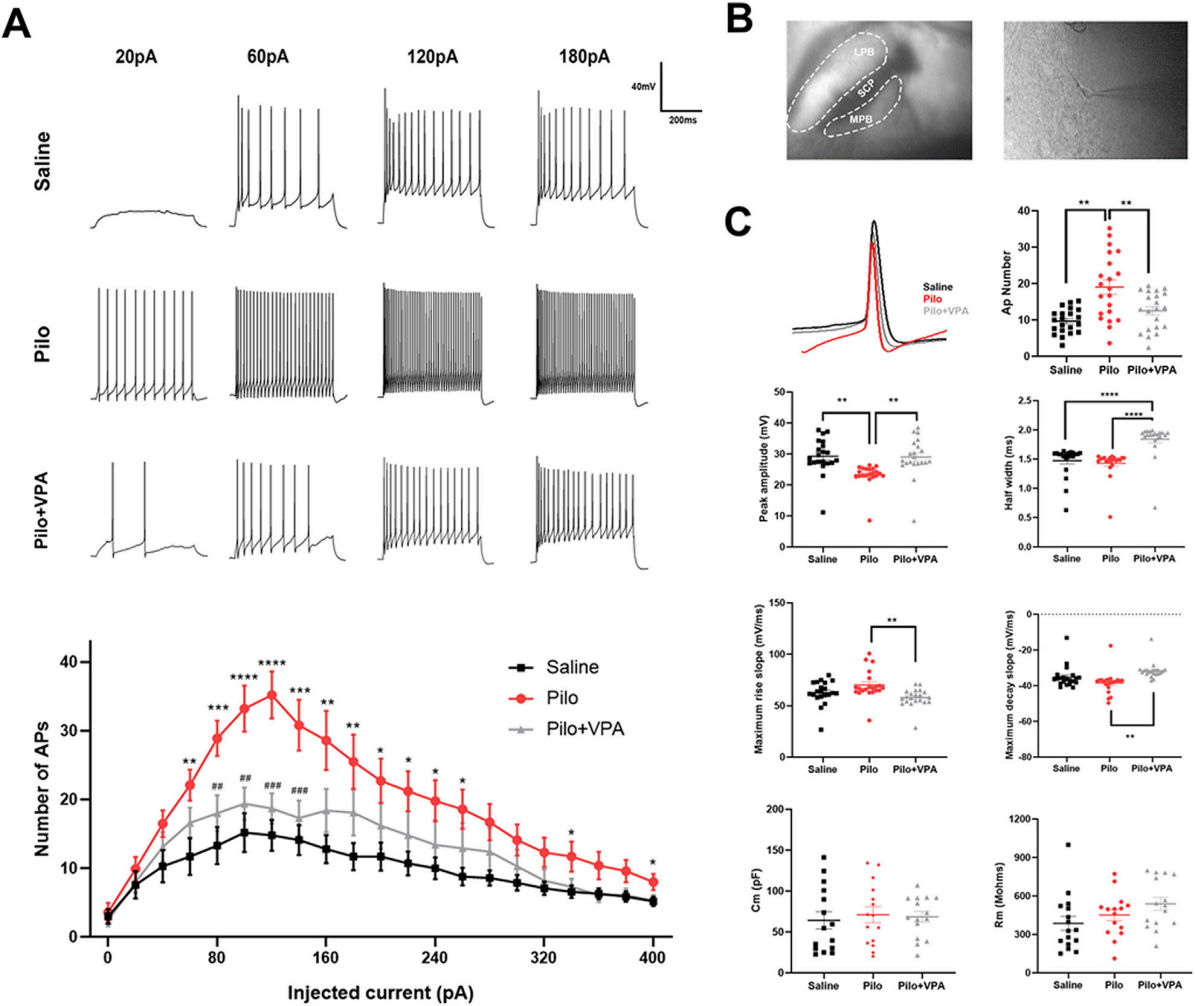
Whole-cell current clamp recordings of the medial parabrachial nucleus (MPB) neurons in brain slices from mice injected with saline, pilocarpine (Pilo), or Pilo + valproic acid (VPA) **(A)** MPB neurons were electrically stimulated with 500-m current injections from 20 to 400 pA in 20 pA increments **(B)** Representative MPB coronal brain slices showing the location of whole-cell current clamp recordings **(C)** Action potential (AP) recordings of MPB neurons in the saline (black), Pilo (red), and Pilo + VPA (gray) groups. AP number, amplitude, half-width, maximum rising slope, maximum falling slope, as well as neuronal membrane capacitance (C_m_) and membrane resistance (R_m_) in the physiological saline, Pilo, and Pilo + VPA groups are indicated. Neuronal firing frequency between groups was compared using the independent sample Student’s t-test. Statistical significance of differences is indicated as follows: *P < 0.05, **P < 0.01, ***P < 0.001. Data are shown as the mean ± standard error of the mean.

Pilocarpine was injected intraperitoneally, and the modeling success rate was 63%. To assess active neuronal responses, we used the current clamp technique to evoke action potentials in MPB neurons in brain slices from animals of the three experimental groups (saline, pilocarpine, and pilocarpine + VPA). Specifically, we determined action potential firing frequency, half-width, peak amplitude, maximum rising slope/maximum falling slope, and action potential threshold. Action potentials were evoked in MPB neurons in response to 500 m current steps of 0–400 pA at 20 pA increments. We found that the membrane capacitance and membrane resistance of neurons in the pilocarpine and pilocarpine + VPA groups were not significantly different from those in the saline group. This finding suggests that the membrane properties of MPB neurons were not significantly altered in the chronic phase of pilocarpine-induced epilepsy and, therefore, have no effect on active responses during current clamp recordings (Figure 1). In addition, more action potentials were recorded in the pilocarpine injection group than in the saline group. After 28 days of VPA administration by gavage, the number of action potentials generated by MPB neurons in mice in the pilocarpine group significantly decreased compared with the saline group. These results suggest that pilocarpine-induced epilepsy increases neuronal excitability within the MPB during the chronic phase.

### 3.2 Pilocarpine increases GFAP protein expression in the MPB during the chronic phase of epilepsy in mice

**FIGURE 2:**
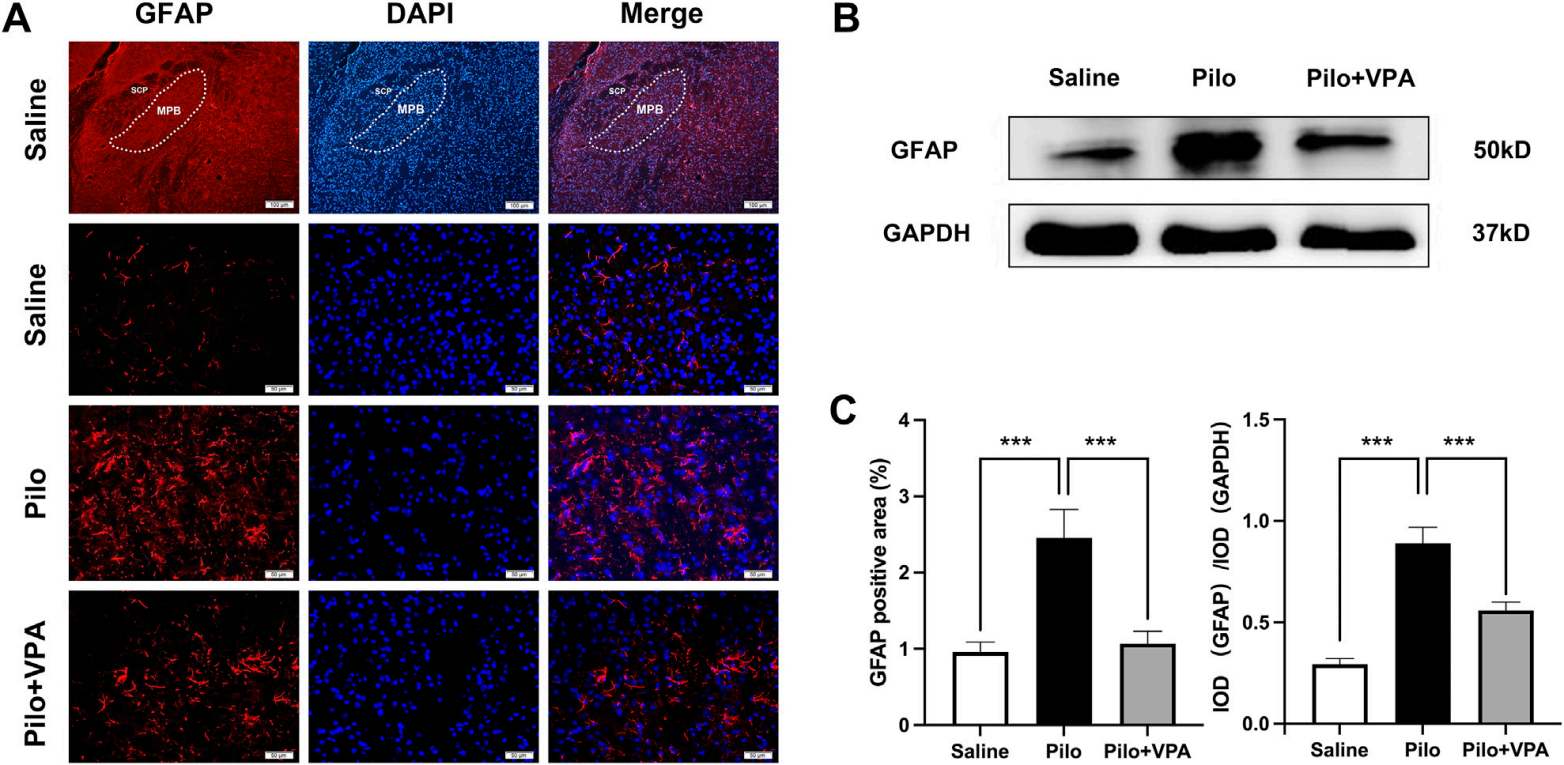
Pilocarpine significantly increases glial fibrillary acidic protein (GFAP) expression in the chronic phase of epilepsy in mice **(A)** Immunofluorescence staining of GFAP in the medial parabrachial nucleus (MPB) in slices obtained from animals in the saline, pilocarpine (Pilo), and Pilo + valproic acid (VPA) groups. The GFAP protein is stained in red, and 4′,6-diamidino-2-phenylindole-stained nuclei are stained in blue **(B)** Western blot detection of GFAP expression in the three experimental groups [**(C)**, left] Statistical analysis of GFAP expression assessed using immunofluorescence staining [**(C)**, right] Statistical analysis of GFAP expression assessed using Western blot. Statistical analysis of GFAP expression was assessed using Student’s t-test and one-way ANOVA. Statistical significance of differences is indicated as follows: ***P < 0.001. Data are shown as the mean ± standard error of the mean.

In the immunofluorescence staining experiment, the GFAP-positive staining area was significantly increased in the Pilo group compared to the Saline group (P < 0.001; Figure 2A). Furthermore, the GFAP-positive staining area in the Pilo + VPA group was significantly reduced compared to the Pilo group (P < 0.001; Figure 2A). Specifically, the area of GFAP-positive staining was 0.95 ± 0.53 in the Saline group, 2.45 ± 1.51 in the Pilo group, and 1.07 ± 0.67 in the Pilo + VPA group (Figure 2C, left panel). In the Western blotting experiment, GFAP protein expression was significantly elevated in the Pilo group compared to the Saline group (P < 0.001; Figure 2B). Conversely, GFAP protein expression was significantly decreased in the Pilo + VPA group compared to the Pilo group (P < 0.05; Figure 2B). The semi-quantitative analysis of GFAP in the medial parabrachial nucleus yielded the following results: Saline group: 0.29 ± 0.05; Pilo group: 0.89 ± 0.13; Pilo + VPA group: 0.56 ± 0.06 (Figure 2C, right panel). These results suggest that pilocarpine-induced epilepsy influenced the MPB, while treatment with VPA alleviated this effect.

### 3.3 Pilocarpine increases FosB/ΔFosB protein expression in the MPB during the chronic phase of epilepsy in mice

**FIGURE 3:**
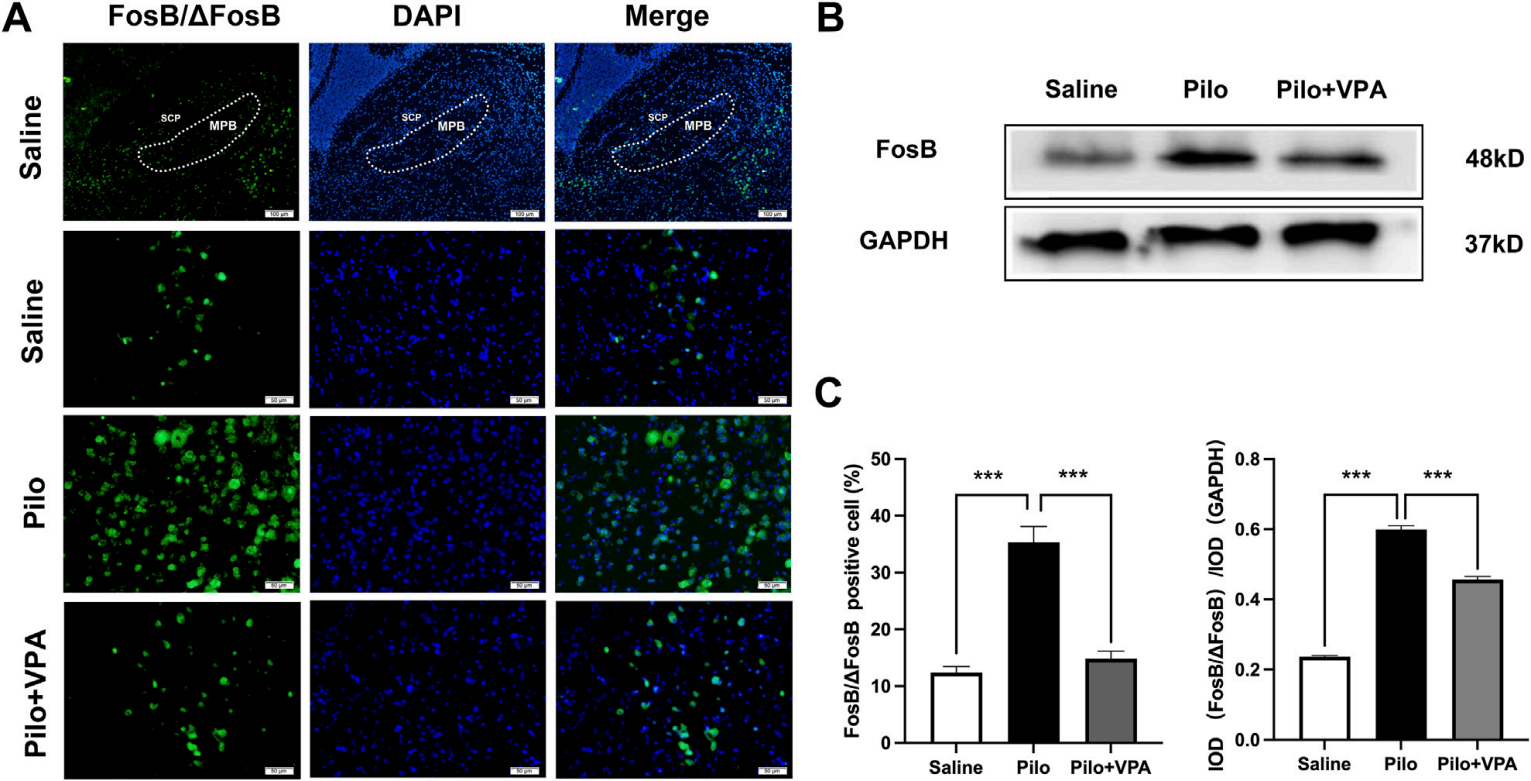
Pilocarpine increases FosB/ΔFosB expression in the chronic phase of epilepsy in mice **(A)** FosB/ΔFosB protein expression in the medial parabrachial nucleus (MPB) in slices obtained from animals in the saline, pilocarpine (Pilo), and Pilo + valproic acid (VPA) groups. The FosB/ΔFosB protein is stained in green, and 4′,6-diamidino-2-phenylindole-stained nuclei are stained in blue **(B)** Western blot detection of FosB/ΔFosB expression in the three groups [**(C)**, left] Statistical analysis of FosB/ΔFosB expression revealed using immunofluorescence staining [**(C)**, right] Statistical analysis of FosB/ΔFosB expression revealed using Western blotting. Statistical analysis of FosB/ΔFosB expression was assessed using Student’s t-test and one-way ANOVA. Statistical significance of differences is indicated as ***P < 0.001. Data are shown as the mean ± standard error of the mean.

FosB/ΔFosB protein expression levels are widely used as markers to identify recently active neurons (Nestler et al., 1999; Nestler, 2015). In the immunofluorescence staining experiment, the number of FosB/ΔFosB-positive cells was significantly increased in the Pilo group compared to the Saline group (P < 0.001; Figure 3A). Furthermore, the Pilo + VPA group exhibited a significant decrease in FosB/ΔFosB-positive cells compared to the Pilo group (P < 0.001; Figure 3A). Specifically, the number of FosB/ΔFosB-positive cells was 12.33 ± 4.10 in the Saline group, 35.33 ± 10.86 in the Pilo group, and 14.82 ± 4.46 in the Pilo + VPA group (Figure 3C, left panel). In the Western blotting experiment, the expression level of ΔFosB protein was significantly elevated in the Pilo group compared to the Saline group (P < 0.0001; Figure 3B). Conversely, the expression level of ΔFosB protein was significantly decreased in the Pilo + VPA group compared to the Pilo group (P < 0.0001; Figure 3B). The semi-quantitative analysis of FosB/ΔFosB in the medial parabrachial nucleus yielded the following results: Saline group: 0.23 ± 0.01; Pilo group: 0.60 ± 0.02; Pilo + VPA group: 0.45 ± 0.02 (Figure 3C, right panel). The results of the immunofluorescence and Western blotting experiments were consistent with those of current clamp recordings, indicating that pilocarpine-induced epilepsy increased neuronal excitability in the MPB.

## 4 Discussion

In the present study, we investigated changes in the excitability of MPB neurons in a mouse chronic epilepsy model using the whole-cell current clamp technique. We further assessed changes in the expression of FosB and GFAP during the chronic phase of epilepsy through immunofluorescence staining and Western blotting. We examined the effects of a 28-day treatment with valproate on neuronal excitability in the MPB and the expression of FosB and GFAP. Our findings indicated increased excitability of MPB neurons and elevated astrocyte reactivity in the chronic epilepsy model. Notably, following treatment with sodium valproate, we observed a marked reduction in both action potential generation and the expression levels of GFAP and FosB proteins.

The role of astrocytes in epilepsy has been extensively investigated, with evidence indicating that astrocyte reactivity, as demonstrated by increased GFAP expression, contributes to neural hyperexcitability in several brain regions, including the hippocampus and thalamus, during chronic temporal lobe epilepsy (TLE) (Garzillo and Mello, 2002; Zhang et al., 2015). In alignment with these findings, our study revealed an elevation in GFAP expression within the medial parabrachial nucleus (MPB) during the chronic phase of epilepsy. This suggests that astrocyte proliferation and activation in this region may play a role in enhancing the excitability of MPB neurons. Previous research has shown that astrocytes modulate neuronal excitability by influencing neurotransmitter metabolism and maintaining electrolyte balance (Verhoog et al., 2020; Perea et al., 2009). Our data support these conclusions by indicating a potential relationship between astrocyte reactivity and MPB neuron hyperexcitability in the context of chronic epilepsy.

We used the whole-cell current clamp technique to record excitability changes in MPB neurons in brain slices from mice in the chronic phase of TLE. Our electrophysiological recordings demonstrated a significant reduction in the action potential threshold in MPB neurons from pilocarpine-induced epilepsy mice compared with that in the saline group. These results suggest that lower stimuli may induce MPB neurons to generate action potential discharges, resulting in the enhanced excitability of MPB neurons. In addition, the threshold and peak amplitude of action potentials after oral administration of VPA were significantly higher in MPB neurons in the pilocarpine + VPA group than in neurons in the pilocarpine group and closer to the levels observed in neurons of the saline group. At the same time, the half-width, maximum rising slope, and maximum falling slope of the action potentials in the pilocarpine group were not significantly different from those in the saline group. This finding suggests that the ion channel properties of MPB neurons and ion concentrations inside and outside the cell membrane were not significantly altered in the pilocarpine group. Therefore, we hypothesized that epilepsy induction enhanced the neuronal excitability of MPB neurons. Notably, action potential firing frequency peaked in the saline and pilocarpine + VPA groups after injection of 100 pA and in the pilocarpine group after injection of 120 pA. At 0–260 pA, the number of action potentials in the pilocarpine group was significantly higher than that in the saline group. Although action potential frequency in the pilocarpine group began to decrease after reaching its peak, the frequency remained consistently higher than that in the saline group, suggesting that the excitability of MPB neurons was significantly higher in the pilocarpine group. Moreover, we observed no significant difference in the number of evoked action potentials between the pilocarpine + VPA and saline groups, suggesting that VPA inhibited the excitability of MPB neurons. However, we observed a significant difference in the firing frequency between neurons from the pilocarpine and pilocarpine + VPA groups, suggesting that VPA was most effective when the neuronal injection current was 80–120 pA. The results of the present study confirmed that the excitability of MPB neurons increases during the chronic phase of epilepsy, suggesting changes in MPB neuronal excitability during TLE. Regarding the potential mechanisms underlying the reduction in AP threshold, we propose the following hypotheses: Enhanced synaptic transmission may also contribute to this process. Epileptic discharges might increase glutamatergic synaptic inputs or decrease GABAergic inhibitory inputs, leading to greater excitatory stimulation of mPBN neurons and thereby lowering the threshold for triggering action potentials. Studies have shown that Sevoflurane depresses neurons in the medial parabrachial nucleus by potentiating postsynaptic GABA_A_ receptors and background potassium channels (Xu et al., 2020). Furthermore, the overall hyperexcitability of the neural network in the epileptic state may further amplify the functional changes in mPBN neurons. Epilepsy is often accompanied by inflammatory responses. Clinically, the serum levels of IL-1β, IL-6, and IL-1Ra are significantly elevated in patients with epilepsy (Uludag et al., 2015). In animal experiments, intraperitoneal injection of pilocarpine can significantly increase the levels of inflammation-related factors such as TNF-α, PEG2, and IL-1β in the hippocampus (Arisi et al., 2015; Vizuete et al., 2017; Vizuete et al., 2018). We found that inflammation leads to the downregulation of inwardly rectifying potassium channels (Kir). Both *in vivo* and *in vitro* studies have shown that local inflammation, particularly interleukin-1β (IL-1β), can downregulate the mRNA and protein expression of Kir4.1 (Zurolo et al., 2012). Kir channels are closely related to the pathogenesis and treatment of epilepsy (Kofuji and Newman, 2004; Olsen and Sontheimer, 2008; Simard and Nedergaard, 2004; Walz, 2000). The Kir4.1 channel is highly expressed in astrocytes, where it helps transport excess K+ ions secreted by excitatory neurons to regions with lower K+ concentrations, thus maintaining neuronal excitability (Kekesi et al., 2017; Larsen and MacAulay, 2014; Nwaobi et al., 2016). It has been confirmed that Kir4.1 expression is reduced in the sclerotic hippocampus of TLE patients (Das et al., 2012; Heuser et al., 2012). In experimental animals, pilocarpine-induced epilepsy results in a significant decrease in Kir4.1 expression, concomitant with a marked increase in GFAP in the hippocampus (Vizuete et al., 2017). Kir4.1 expression is also significantly reduced in NER and Lgi1 genetic epilepsy model rats (Harada et al., 2013; Kinboshi et al., 2019). The significant reduction in Kir4.1 expression impairs astrocytic K^+^ reuptake, causing excessive K^+^ accumulation in the extracellular space, thereby altering the neuronal resting membrane potential and increasing neuronal excitability. Studies have shown that Kir4.1 channels are expressed in the MPB (Wu et al., 2004). Therefore, we hypothesize that epilepsy-induced neuroinflammation may lead to the downregulation of Kir4.1 expression in the MPB of model mice, which in turn increases the activity of glutamatergic neurons in the MPB. The reduction in AP threshold may reflect increased sensitivity of the mPBN within the epileptic network and could be a key mechanism underlying the propagation of generalized seizures.

Furthermore, we utilized immunofluorescence techniques to demonstrate an increased expression of FosB within the medial parabrachial nucleus (MPB). FosB serves as an indicator of neuronal activity, and similarly, previous studies have reported that ΔFosB expression and AP-1 DNA binding are significantly elevated in epileptic animals for up to 1 year. The upregulation of neuronal ΔFosB has been associated with brain regions involved in the generation and propagation of seizures (Morris et al., 2000). Thus, our findings suggest that the increased excitability of MPB neurons may represent a potential mechanism underlying seizure activity. Additionally, astrocytes may contribute to enhanced neuronal excitability through the release of pro-inflammatory factors and the modulation of neurotransmitter metabolism. This abnormal glial response could lead to the hyperactivation of neurons in the MPB region, thereby triggering seizures.

Additionally, the parabrachial nucleus (PB) is a critical component of the brainstem arousal system, comprising glutamatergic, GABAergic, and enkephalinergic neurons. In our study of the parabrachial nucleus (PBN), we specifically focused on the neurophysiological and functional differences between the medial parabrachial nucleus (MPB) and the lateral parabrachial nucleus (LPB). Although both MPB and LPB are components of the PBN, they exhibit significant differences in anatomy and function. Previous studies have shown that the MPB is primarily involved in emotional regulation, autonomic nervous responses, and complex visceral feedback, all of which are closely related to the onset and progression of epilepsy. Notably, the MPB’s role in regulating emotional states and autonomic nervous function may be strongly associated with the neural network changes observed during the chronic phase of epilepsy. In contrast, the LPB is more involved in the processing of sensory information, particularly in responding to sensory stimuli during wakefulness. The activity of the LPB is critical for the regulation of the sleep-wake cycle, arousal states, and sensory information processing. The medial parabrachial nucleus (MPB) plays a significant role in the transition from REM to NREM sleep, with impairments in the MPB associated with increased REM sleep in animal models. Conversely, heightened excitability of the MPB is likely to shorten REM sleep duration and increase susceptibility to seizures. Furthermore, research has shown that sleep deprivation significantly increases seizure frequency in patients with temporal lobe epilepsy (TLE), suggesting a complex interplay between the MPB and sleep regulation.

Meanwhile, the effects of valproate (VPA) have received considerable attention in many studies. As a commonly used medication for treating temporal lobe epilepsy (TLE), VPA has been shown to significantly improve sleep quality and reduce seizure frequency in patients. Chronic treatment with valproate (VPA) is widely recognized for its anticonvulsant properties, particularly in the context of epilepsy management. Previous studies have demonstrated that VPA can significantly reduce both the frequency and severity of seizures in various animal models of epilepsy (Cognato et al., 2007; Deng et al., 2017; Turski et al., 1987). It is thought to exert its effects primarily through enhancing GABAergic activity and stabilizing synaptic transmission, which are crucial mechanisms in controlling seizure activity and preventing epileptic behaviors. Chronic VPA treatment did not affect the expression levels of c-Fos protein in the PBN (Pal et al., 2022). Literature has reported that VPA not only suppresses seizures but also enhances sleep quality (Tallavajhula and Slater, 2012). In this study, we investigated changes in neuronal excitability in the medial parabrachial nucleus (MPB) during the chronic phase of epilepsy in the same TLE mouse model. The results indicate that VPA treatment reduces MPB neuronal excitability, potentially exerting its protective effects by inhibiting the overexpression of GFAP. VPA may disrupt the sleep architecture of patients with epilepsy by increasing NREM phase 1 and decreasing the REM phase, while also stabilizing and improving sleep structure (Zhang et al., 2014).

Our study has several limitations. First, as an *in vitro* investigation primarily based on mouse models, the findings require cautious interpretation and application. The relatively small sample size also limits the generalizability and reliability of the results, highlighting the need for larger-scale studies to ensure robustness and representativeness. While our research focused on the response of the medial parabrachial nucleus (MPB) to valproate (VPA), we observed significant input from the bed nucleus of the stria terminalis (BNST) to the MPB in the Dravet syndrome mouse model (Yan et al., 2021). This suggests that VPA’s effects may not solely depend on its direct action on the MPB; rather, the BNST may regulate MPB activity, implying an indirect modulatory mechanism. However, this hypothesis remains unconfirmed and requires further investigation to better understand the neurobiological mechanisms underlying VPA’s effects. Additionally, our study did not distinguish between excitatory and inhibitory neurons due to experimental design constraints. Immediate early genes (IEGs), used as markers of neuronal activity, are also expressed in non-neuronal cells such as glial cells, presenting a potential limitation. We used DAPI for nuclear staining to label all cell populations; however, as a non-specific marker, it does not differentiate neuronal from non-neuronal cells. To address this limitation, future studies will validate the neuronal specificity of IEG expression using co-staining with markers such as NeuN. This will clarify the overlap between IEG expression and neuronal populations, improving the accuracy of our conclusions. These additional experiments will strengthen our findings, enhance understanding of neuronal homeostasis at the molecular level, and address current limitations, laying a stronger foundation for future studies on VPA’s effects and its potential implications for treating epilepsy and sleep disorders.

In summary, our research indicates that in a chronic epilepsy model, the excitability of medial parabrachial nucleus (MPB) neurons is significantly increased, accompanied by enhanced reactivity of astrocytes. This change predisposes individuals to seizures, potentially forming a vicious cycle that contributes to sleep disorders in temporal lobe epilepsy. The antiepileptic drug sodium valproate (VPA) exerts its effects by inhibiting the excitability of MPB neurons through direct or indirect pathways. These findings deepen our understanding of the pathogenesis of temporal lobe epilepsy and provide new perspectives and strategies for its treatment.

## Data Availability

The raw data supporting the conclusions of this article will be made available by the authors, without undue reservation.

## Abbreviations

ACSF, Artificial cerebrospinal fluid; AP, Action potential; GFAP, Glial fibrillary acidic protein; IEDs, Interictal epileptic discharges; MPB, Medial parabrachial nucleus; NREM, Non-rapid eye movement; PACAP, Pituitary adenylate cyclase activating polypeptide; PB, Parabrachial nucleus; PBS, Phosphate-buffered saline; REM, Rapid eye movement; SE, Status epilepticus; TLE, Temporal lobe epilepsy; VPA, Valproic acid.
